# STAKEHOLDER PERSPECTIVES ON IMPLEMENTATION OF INTERNET-DELIVERED COGNITIVE BEHAVIOUR THERAPY IN PHYSICAL MEDICINE REHABILITATION SETTING USING THE CONSOLIDATED FRAMEWORK FOR IMPLEMENTATION RESEARCH

**DOI:** 10.2340/jrm.v57.40898

**Published:** 2025-01-14

**Authors:** Swati MEHTA, Ujjoyinee BARUA, Marcie NUGENT, Kevin HANSEN, Luvish SONDHI, Randy UPPER, Dalton WOLFE, Eldon LOH, Keith SEQUEIRA, Robert TEASELL, Heather D. HADJISTAVROPOULOS

**Affiliations:** 1 Lawson Health Research Institute, London, ON; 2 Department of Psychology, University of Regina, Regina, SK, Canada

**Keywords:** physical rehabilitation, internet delivered cognitive behaviour therapy, implementation

## Abstract

**Introduction:**

Despite the growing evidence for the effects of tailored internet-delivered cognitive behaviour therapy (ICBT) programmes for those receiving physical rehabilitation, there is a lack of implementation of these programmes in a clinical or community setting. The aim of the current study was to evaluate barriers and facilitators of implementing an ICBT programme into a physical medicine rehabilitation setting.

**Methods:**

Stakeholders with expertise in physical medicine rehabilitation were recruited (*n* = 25) including: 16 clinicians, 4 administrators, 3 persons with lived experience, and 2 care partners. Individual semi-structured interviews were conducted based on the domains of the Consolidated Framework for Implementation Research (CFIR). Transcripts were analysed using a positivist approach, using deductive thematic content analysis. Themes were coded based on the domains of CFIR.

**Results:**

Facilitators for implementation primarily fell under intervention characteristics including relative advantage, strong evidence and quality, and design quality. Perceived barriers for implementation were identified in the inner setting including leadership engagement, culture, and available resources.

**Conclusions:**

The results from the current study provide insight on the factors that may contribute towards successful implementation of an ICBT programme in a physical medicine setting.

## LAY ABSTRACT

Online mental health programmes designed specifically for people in physical rehabilitation have shown promising results. However, these programmes are not widely used in clinics or community settings. This study aimed to understand what helps or hinders the adoption of these online therapy programmes in rehabilitation settings. We conducted an interview among 16 healthcare professionals, 4 administrators, 3 individuals with lived experience, and 2 family caregivers concerning their thoughts on implementing a tailored online mental health programme in a physical rehabilitation setting. Results from the interviews found that factors that help adoption of the programme include: mental health benefits, well-supported by evidence, and their user-friendly design. However, challenges such as limited leadership support, workplace culture, and lack of resources were seen as obstacles for adopting these programmes. This study highlights what needs to be in place for adoption of these programmes to succeed in rehabilitation settings, offering guidance for future efforts to make them more accessible and widely used.

Depression and its associated disorders among people with spinal cord injury (SCI) are the most frequent form of psychological distress, with studies reporting a 48% prevalence rate of clinically significant depressive symptoms (1). Elevated levels of anxiety have also been found in the SCI population with prevalence rates ranging from 15% to 32% (2). Psychosocial management approaches that target emotional distress and promote well-being are integral in improving quality of life (3). Reviews examining the effectiveness of psychosocial interventions based on cognitive behaviour therapy among those with SCI found moderate (≥ 0.5) to large (≥ 0.8) effects on measures of depression and anxiety as well as assertiveness, coping, self-efficacy, and quality (3, 4).

Among psychological interventions, cognitive behaviour therapy (CBT) has been extensively studied and has a strong evidence base demonstrating long-term effects for improving individuals’ psychological health through modifying unhelpful emotional, behavioural, and cognitive responses (5). In SCI populations, CBT focuses on learning SCI-specific coping strategies to manage stressful stimuli and develop strategies to help modify an individual’s response to the stressor (6). Several barriers, such as financial strain, geographical barriers, and poor functional ability, however, can reduce access to mental health programmes among those with SCI (7). As approximately 70% of people with SCI report already using a computer and 94% of these report accessing the internet (8), online delivery of psychosocial interventions may provide a cost-effective and accessible solution to these barriers.

Internet-delivered CBT (ICBT) provides online structured self-help modules based on the core principles of CBT. These modules can be delivered in a self-directed format or in combination with weekly support from a registered mental health professional (i.e., social worker, psychotherapist, psychologist). ICBT has previously been shown to have an advantage over traditional face-to-face psychotherapy as it offers greater consistency and quality of information presented to clients (9). Other reported advantages of ICBT over face-to-face programmes include being more cost-effective (10, 11), increased accessibility (12), privacy, and lowering mental health stigma (13). The effectiveness of ICBT in improving symptoms of depression and anxiety in various somatic health conditions and underserviced populations, including older adults (14), cancer survivors (15, 16), and individuals with acute coronary events (17), have also been reported. A meta-analysis of 20 studies investigating the effect of ICBT among those with chronic health conditions found that in a pooled sample of over 2,400 participants, ICBT significantly improved overall anxiety (standard difference in mean (SDM) = 0.34 ± 0.04, 0.18–0.49, *p* < 0.001) and depression (SDM = 0.33 ± 0.04, 0.17–0.49, *p* < 0.001) (18). Additionally, a systematic review of 22 studies examining the effect of ICBT on chronic pain found small (≥ 0.2) to moderate (≥ 0.5) effects of ICBT on outcomes of pain interference, disability, pain intensity, and pain catastrophizing when compared with the waitlist control group (19). Thus, these studies suggests that clinician-guided ICBT is a safe and effective alternative to traditional face-to-face interventions in populations with chronic health conditions.

A limited number of studies to date have examined determinants for implementing ICBT. These have primarily been reported in community settings such as primary care and psychiatric facilities (20, 24). Unlike those in the general psychiatric populations, individuals undergoing physical rehabilitation are likely to experience physical and cognitive impairments that may result in differing challenges to accessing ICBT. Furthermore, critics of technology-based mental health interventions argue that these interventions fail to include the perspectives of stakeholders (25). Thus, examining barriers and facilitators of implementing the ICBT programme in a physical medicine and rehabilitation (PMR) setting is warranted to better understand the practicality, acceptability, and scalability of such a delivery model.

Developing implementation strategies for the successful adoption of eHealth interventions requires the use of theoretical frameworks (26, 27). The current study used the Consolidated Framework for Implementation Research (CFIR) based on its comprehensive nature (28). While several evidence-based implementation frameworks can be used to guide implementation design and evaluation, CFIR is one of the most frequently cited in the implementation literature. Due to its multifaceted nature, it allows for evaluation of various components of interventions to guide changes in models of care. Additionally, CFIR provides freely available data analytics tools such as interview guides and coding templates to enhance the research process (27). CFIR assesses multiple factors related to implementation success (28) and is composed of 5 main domains: (*i*) intervention characteristics (e.g., evidence, advantages, complexity, quality, cost); (*ii*) outer setting (e.g., patient needs, networks, policies); (*iii*) inner setting (e.g., structure, communication, culture); (*iv*) characteristics of individuals (e.g., knowledge of intervention, self-efficacy, individual stage of change); and (*v*) process (e.g., planning, engaging, conducting, reflecting, evaluating) (28). CFIR has been used in several implementation studies to evaluate factors that may act as facilitators and/or barriers in a variety of conditions (29–30). The current study aims to evaluate barriers and facilitators of implementing an ICBT programme in a PMR setting using the CFIR framework through a qualitative approach.

## METHODS

### Study design

A qualitative approach was used to evaluate the facilitators and barriers of implementing a tailored ICBT programme for those undergoing physical rehabilitation (31, 32). The study was examined using a post-positivist paradigm, recognizing that complete objectivity is often unachievable but striving to be as neutral and objective as possible. Semi-structured interviews were conducted with both stakeholders and programme end-users. The study was approved by the research ethics board at Western University.

### Participants and recruitment

Participants with a background in physical rehabilitation including clinicians, administrators, persons with lived experience, and care partners were included. Participants were excluded if they had cognitive impairments or were unable to communicate in English. Participants were recruited across Canada through physical medicine rehabilitation facilities and community groups (Spinal Cord Injury Ontario, March of Dimes), advertisements on social media (e.g., Twitter, Facebook) and internal rehabilitation organizational email listservs, and institutional patient-oriented research committees.

### Procedure

Once the research coordinator had gained participants’ consent, they were asked to complete background questionnaires using the Research Electronic Data Capture (REDCap) web application. All participants completed questionnaires that assessed demographic factors such as age, sex, and location of residence. Location of residence was defined as: large city (population over 200,000); small to medium city (population 10,000 to 200,000), or rural area/farm (population under 10,000). Hospital administrators and clinicians were also asked to indicate the number of years of experience they had working in a rehabilitation setting. Those with lived experience (i.e., having an SCI) were asked for injury-related information including severity and level of spinal cord injury, and length of time since injury occurred. Once the background questionnaires were completed, participants received a brief background on ICBT and a copy of the interview questions in advance (Appendix A). Participants were asked to schedule an appointment for an online semi-structured video interview. All interviews were conducted by 2 graduate students (LS, male, 23 years old, Master’s student; UB, female, 25 years old, PhD student) with a previous background in qualitative research. All interviews lasted between 20 and 70 min and were conducted through the WebEx video conferencing software. Interviews were recorded through a digital voice recorder and transcribed verbatim by the graduate students. Participants who did not want voice recording were offered instead that notes be taken by the interviewer. However, all participants allowed voice recording.

### Interview

Guided by the CFIR Interview Guide Tool, a semi-structured interview guide was developed that utilized pre-defined questions designed to capture potentially unexpected responses, which often occur in qualitative research studies (33, 34). Appendix A provides a guide for example questions used during the interview. Questions were adapted based on the role of the participant. For example the question: “What kinds of changes or alterations do you think you will need to make to ICBT so it will work effectively in your setting?” was changed to “What kinds of changes do you think we will need to make to ICBT so it will meet your or your loved ones’ needs?” when speaking to those with lived experience or care-partners.

### Analysis

Transcripts were analysed using a deductive approach based on the existing CFIR framework. Deductive thematic analysis aims to identify patterns within and across data in relation to participants’ lived experiences and perspectives and seeks to understand what participants think, feel, and do (31, 32). Data analysis was performed by 3 members of the research team SM, LS, and UB. The first member (SM) is a scientist and an assistant professor with previous experience in qualitative research. The second member (LS) is a first-year graduate student with previous experience in qualitative research. Lastly, UB is a second-year graduate student with previous experience in qualitative research. The research team needed to consider how their interactions with the participants and interpretation of the data might be influenced by their own background, experiences and assumptions. The team also reflected on how potential power dynamics may influence participants’ willingness to share their experiences. Analysis was performed in a stepwise iterative process by SM and LS, with each transcript being initially read in its entirety before scoring. The CFIR framework domains (i.e. intervention characteristics, individual characteristics, etc.) were used in the initial coding process as primary themes (28). A description of the themes and subthemes in the CFIR framework can be found at: https://cfirguide.org/wp-content/uploads/2019/08/cfirconstructs.pdf. The coding was discussed between raters to ensure relevance to the specific domains. Next, coding was further broken down and classified into subthemes based on CFIR subconstructs under each domain. For example, codes under the theme “Intervention characteristics” were further classified into sub-themes such as “innovation”, “evidence strength & quality”, “relative advantage”. Next, coders classified semantic units as facilitators or barriers within each node or sub-node. The final coding was reviewed and agreed upon by SM, LS, and UB. The large amount of data was organized and managed using the software program NVivo version 12 (QSR International, Burlington, MA, USA). Representative quotations with a breakdown of the contributions from each group to the relevant CFIR domain are presented in the subsequent results to increase transparency of the interpretations (Table I). The number of unique statements from all participants which supported that a construct from the CFIR framework was present was calculated based on a frequency score (Table I).

**Table I:** Overview of the CFIR constructs and related frequencies of unique statements

CFIR Construct	Facilitators				Barriers				Representative comments

	Clinicians	Administrators	Persons with lived experience	Care partners	Clinicians	Admini-strators	Persons with lived experience	Care partners	
Intervention characteristics									
Innovation									
Evidence strength and quality	19	3	4	2					“The programme has a strong evidence based with multiple studies reporting on the effectiveness” (Clinician) “My husband went through the programme and really benefited from it, there was a change in his outlook and sense of purpose” (Care partner)
Relative advantage	16	4	3	2					“It has the ability to service a larger audience that may experience barriers to in person care” (Administrator) “I like not having to drive 3 hours for a 1 hour appointment” (Person with lived experience)
Adaptability					5				“Patients with neurological conditions have greater needs and those participating may not have the skills needed to engage on their own” (Clinician)
Trialability									
Complexity					3		1		“The platform and reading material may be hard for people that have severe cognitive impairments to follow” (Clinician) “I’m not great with computers and type really slow” (Person with lived experience)
Design quality and packaging	6		2	1					“It’s nicely structured and flows well. The case vignettes really help to normalize many concerns” (Clinician) “I liked that it was structured in a linear format where the first lesson builds on to the next. This way I was guided along the way and I wasn’t just left alone to figure things out” (Person with lived experience)
Cost	14	2				1	1	1	“There’s limited funding to integrate mental health care into inpatient rehabilitation. We have a psychiatrist come once a month to complete assessments” (Administrator) “I don’t think my insurance would cover this programme” (Person with lived experience)
Total	55	9	9	5	8	1	2	1	
Outer setting									
Needs and resources	17	4	2	2					“My clients get stuck on a wait list over a year to get in to see a psychologist at the hospital. Having an online programme would open up possible resources for clients” (Clinician) “There aren’t many specialized mental health services. We’ve tried private counselling but it didn’t really help him” (Care partner)
Cosmopolitanism									
Peer pressure									
External policy and incentives									
Total	17	4	2	2					
Inner setting Structural characteristics					7	2			“The way the hospital is structured, implementing anything that involves changes to the electronic referral process is challenging and can take forever” (Clinician)
Networks and communications									
Relative priority	21	4	4	2					“Mental health is so important for those undergoing rehabilitation. Getting them motivated to do the exercises I prescribe can be challenging. There’s a real need to implement programmes like this” (Clinician) “Having an online resource that’s tailored to my needs is important” (Person with lived experience)
Culture					5				“At our centre, there are a lot of clinicians that like how things are being done and don’t want to change anything” (Clinician)
Leadership engagement					12				“Managers say they want a programme like this, but don’t want to commit to anything” (Clinician)
Available resources					16	6			“We just don’t have any additional funding to support this at our centre” (Administrator)
Implementation climate									
Total	21	4	4	2	40	8			
Individual characteristics									
Knowledge and beliefs about the innovation	15	4	4	2	4				“There is a strong evidence base for ICBT programmes with several meta-analysis supporting its use” (Clinician) “The program me really changed the way my husband views life. I wish this could be provided to more people” (Care partner)
Self-efficacy							2		“I don’t know if I’ll be able to use the website on my own. It’s hard for me to keep track of my appointments and need someone to help organize everything” (Person with lived experience)
Individual stage of change					5	3			“It’s hard to get some of the clinicians to adopt new procedures” (Administrator)
Individual identification with organization									
Other personal attributes									
Total	15	4	4	2	9	3	2		

## RESULTS

### Participant background

All participants consenting to the study completed the interview. Twenty-five stakeholders participated in the study, including: clinicians: nurses (*n* = 2), occupational therapist (*n* = 2), physiatrist (*n* = 3), physiotherapist (*n* = 1), psychologists (*n* = 2), social workers (*n* = 6); hospital administrators (*n* = 2); community administrators (*n* = 2); persons with lived experience (*n* = 3); and care partners (*n* = 2). Previous experience with and/or knowledge of ICBT was reported by 4 social workers, 1 occupational therapist, 3 physiatrists, 1 hospital administrator, 2 community administrators, 2 persons with lived experience, and 2 care partners. Overall, the majority of respondents reported being women (72%). The average age of persons with lived experience was 52.3 years, while care partners were on average 49.5 years old. Care partners were all females while, those with lived experience were predominantly male (66.7%). Two individuals reported sustaining an incomplete spinal cord injury, while 1 had complete SCI. Level of injury was as follows: 1 cervical, and 2 thoracic with an average time since injury of 14.3 years. Most participants reported residing in a large city (76%), 16% were located in a small city; and 8% in a rural area/farm. Clinicians were primarily in outpatient (37.5%) and community settings (31.2%), while a smaller percentage worked in an inpatient (18.8%) or acute (12.5%) setting. Clinicians had an average of 12.6 years of experience in rehabilitation.

### Intervention characteristics

*Facilitators.* Evidence *strength and quality* was supported by 28 unique statements among the participants. The following facilitators were identified under this construct: (*i*) Empirical evidence to support the intervention. This was seen among clinicians and administrators who stated that they were knowledgeable regarding the empirical evidence for the ICBT programme. (*ii*) Awareness of government supported ICBT programmes. Stakeholders stated that they were aware of government-funded ICBT programmes for the general population, indicating that these programmes were likely to be effective and supported by community experts, thus facilitating stakeholders’ acceptance of the programme. (*iii*) First-hand clinical experience of effectiveness. Clinicians stated that they had previously referred patients to the SCI ICBT programme and found it to improve patients’ mental health:

I currently use CBT in my practice servicing those with an SCI. There is a strong evidence base for its use to manage mental health related outcomes. I find patients like the structure and limited time frame compared to other forms of psychotherapy.

(*iv*) Lived experience of effectiveness (*n* = 3). Persons with lived experience and care partners with experience of the programme described the benefits to their own or their partner’s overall well-being:

I really feel useful again. For so many years I felt like I was treading water, now I feel that I have purpose. Amazing how the ICBT course has changed my life for the positive. So many fantastic friendships have been formed and I’m learning all the time.

*Relative advantage* was supported by 25 unique statements. Under this category, ICBT’s potential to overcome multiple barriers to face-to-face therapy was emphasized as the most common facilitator for implementing ICBT. Participants described the relative advantage of providing care through virtual communications compared with face-to-face. From the clinician perspective, allowing patients to access care in the convenience of their home, without having to worry about wait-times and travelling to appointments, was a commonly stated facilitator. Similarly, among those with lived experience and care partners, advantages to ICBT included being able to engage in the programming at their convenience, i.e., time and location. Additionally, due to the structure of the programme, participants stated that working on the material in short increments allows them to better digest the material and not feel overwhelmed receiving too much information all at once:

It would be nice to not have to drive an hour to the clinic and worry about cancelling because my symptoms flare up. I can do it from home, in short intervals. My wife can help me through the online material, and we can learn things together.

I don’t have to depend on other people’s time to engage in the programme. I can do it on my own when I have breaks.

*Design quality and packaging* was supported by 9 unique statements. Facilitators under this construct included: (*i*) Design of the programme. Participants stated that the manualized design of the programme allowed patients to complete the programme through a self-directed approach, without requiring much guidance. Clinicians reported on the ease of use of the programme and website as well as the structure. (*ii*) Content of the programme. This was seen among clinicians and those with lived experience. Clinicians stated that the content of the programme aligned with the needs of the population. Both clinicians and those with lived experience described how importantly the case vignettes helped to normalize some of their experiences. (*iii*) Guided approach. Participants stated that having weekly guidance was an important facilitator because it allowed them to practise skills and have a sense of accountability and motivation for completing the programme.

*Cost* was supported by 16 unique statements. The primary facilitator under this category was relative cost per patient serviced. Clinicians and administrators stated that because the programme can be delivered through self-direct online modules with brief weekly check-ins of approximately 15–20 min per session per patient, it has a lower cost of programme delivery compared with face-to-face programmes, which requires 40–60 min per session per patient. This allows clinicians to serve the same number of people with lower cost burden to the organization.

*Barriers. Adaptability* was supported by 5 unique statements. The barriers for adaptability included the highly standardized programme. This was related to concerns about the adaptability of the standardized programme for those with greater mental health needs who may require increased flexibility in terms of weekly guidance. Some clinicians were concerned that patients would not receive a response to enquiries until check-in times. They were unsure whether strategies for mitigating crisis interventions among those living in more remote areas would be adequate. Another barrier under adaptability was the potential lack of ability to access the programme among those with dexterity issues. A participant raised concerned about the ability for individuals with dexterity issues to be able to complete homework assignments that require typing on the computer, thus stating that without allowing for adaptive technology to overcome this issue, this may be a barrier for engaging those with higher level of injury:

Will their caregiver need to be with them all the time if they have issues with dexterity?

*Complexity* was supported by 4 unique statements. Barriers to complexity included concerns related to engaging participants that lack comfort with technology. A limited number of stakeholders described some concerns related to the potential complexity of using the online platform and messaging programmes. Those with limited digital literacy or lack of comfort with computers reported the number of steps to log in, get into the lessons, and engage online to be difficult and complex:

I’m not great with computers and type really slow.

*Cost* was supported by 3 unique statements. Barriers to cost included lack of mental health funding. An administrator stated that there is already a lack of funding to support mental health in current rehabilitation programmes. Thus, without changes in health policy or exploration of alternative revenue streams, the implementation of the programme is not likely. Another barrier related to cost of the programme was lack of awareness regarding insurance coverage. Some concerns were raised related to how the costs for an online programme will be billed. Those with lived experience were not sure if the programme was in a community setting that their insurance would cover it.

### Outer setting characteristics

*Facilitators. Patient needs and resources* was supported by 25 unique statements. Facilitators in this section included: (*i*) Identified need for the programme. The need for the programme was commonly cited across all stakeholders interviewed. Participants stated that those in the community experience distress, including issues with adjustment after the injury, developing a new sense of self identity, and re-integrating into the community. Thus, aligning the ICBT programme to meet the needs of the specific population was cited as a facilitator for implementation compared with implementing non-tailored general programmes. Characteristics of tailored programmes include those that are co-designed with knowledge users to ensure their needs are met. (*ii*) Gap in access to resources. Stakeholders stated that though there is great need for mental health programmes, there is a lack of access to these resources. This current lack of access to tailored and specialized programmes serves as a facilitator because, once implemented, the programme would service the gap in patient needs for accessing mental health services:

My clients get stuck on a wait list for over a year to get in to see a psychologist at the hospital, while most don’t qualify to have their needs met.

### Inner setting characteristics

*Facilitators. Relative priority* was supported by 31 unique statements. The primary facilitator for this construct is the current climate for implementing mental health programmes. Stakeholders across the categories reported that providing access to mental health service for patients undergoing physical rehabilitation was a current priority. They described the importance of mental health education and access to resources across the continuum of care, starting with resources for grief and acceptance during the early stages, through to coping and adjustment to life stresses as patients age with a physical disability. The current climate calls for the need for a more holistic approach to rehabilitation that incorporates not just physical and cognitive well-being but also emotional well-being.

*Barriers. Leadership engagement* was supported by 12 unique statements. Barriers under this construct included lack of strategic planning among leadership. Despite the resounding feedback regarding access to ICBT as a priority, participants reported on the lack of engagement among leadership to develop a plan to implement the programme. Participants stated that leadership at their centres acknowledged that ICBT is a relative priority; however, leadership did not follow up with specific plans for adoption. Thus, the lack of concrete plans for potential future implementation led by the leadership team was seen as a barrier.

*Available resources* was supported by 22 unique statements. Barriers in this category included: (*i*) Lack of clinician time to raise awareness. Both administrators and clinicians reported that due to current high caseloads resulting in an unmanageable workload, it was difficult to find the time to educate patients about the programme and engage in the referral process. Thus, lack of time for clinicians to raise awareness of the programme and educate patients concerning the potential benefits acts as a barrier to implementation. (*ii*) Lack of access to funds to implement the programme. Stakeholders stated that lack of funding for mental health resources within their organization limits their ability to implement the programme. Thus, lack of funding for the programme may serve as a barrier for implementation through a sustainable approach:

We don’t have time to answer patients’ questions about ICBT or work on how we can recruit patients. I am an MSW with experience delivering psychotherapy, but all my time gets used up with non-therapeutic activities.

There’s limited funding to integrate mental health care into inpatient rehabilitation. At this time, we have a psychiatrist come once a month to complete assessments.

*Culture* was supported by 5 unique statements. This construct served as a barrier primarily due to a culture among clinicians to resist change. Clinicians reported that their centres included strong personalities who did not want things to change. Implementation of a new programme requires clinicians to learn and implement new care pathways. However, stakeholders stated that many clinicians are resistant to changing current practices. Participants related this to lack of leadership in providing clear goals as well as incentives for behaviour change to occur. Thus, without engagement of leadership and incorporation of incentives, the existing culture to resist change among some clinicians may serve as a barrier for implementation of the programme.

*Structural characteristics* was supported by 9 unique statements. These statements included barriers such as current structure of the organization. Stakeholders described the hierarchical structure of their organization, often citing lack of ability to engage in change with leadership. Participants stated that they would be interested in helping to champion the programme within their organization; however, their voices are often not represented in the decision-making process. Thus, the top-down hierarchical approach in their current setting serves as a structural barrier for implementation of the program. Another barrier related to structural characteristics was hospital policy on technology adoption. Adoption of technology as a barrier was represented in 2 ways. First, participants stated that changing the electronic referral processes can be challenging to implement when working with the hospital IT team. Thus, if clinicians wanted to refer to a community-based ICBT programme, they would have to work with hospital IT teams to incorporate the referral process in a patient’s electronic charts. Additionally, implementation of an ICBT programme within the hospital setting involves strenuous security and privacy assessments. The existing process for approving new programmes among hospital settings was reported to be extremely lengthy and often resource intensive, thus serving as a structural barrier to implementation.

### Individuals’ characteristics

*Facilitators. Knowledge and belief about the intervention* was supported by 25 unique statements. Most stakeholders reported that the intervention would result in improvement of care for those with an SCI. They felt that this would be an evidence-based service that could help bridge the gap in lack of care. Participants reported that there is a need for more virtual care to meet the needs of the population.

*Barriers. Knowledge and belief about the intervention* was supported by 4 unique statements. A small number of clinicians, on the other hand, reported that they were not comfortable with referring patients to online programmes. The clinicians stated that this population needs more intensive care and the online programme would not be as effective as face-to-face treatment. Thus, their lack of knowledge regarding the intervention and its ability to meet the needs of the population may serve as a barrier to implementation:

I don’t think having it online will result in the same therapeutic alliance as face to face, where you can have more of a personal connection with patients and get body language.

*Individual stage of change* was supported by 8 unique statements. Clinicians and administrators reported resistance from some staff on changing how the mental health service is delivered. They stated that implementation would involve changes in their referral and recruitment practices and some clinicians may find it inconvenient. Additionally, clinicians may not feel that they have the skills to deliver the programme in their setting.

*Self-efficacy* was supported by 2 unique statements. Persons with lived experience cited that lack of self-efficacy in engaging in an online programme may be an important barrier to completing the programme. One participant stated that they might not have the skills to complete the programme on their own. Another participant stated that, due to the more self-directed nature of the programme, some participants may not have the discipline to engage consistently.

## DISCUSSION

The current qualitative study utilized interviews following the CFIR conceptual framework to identify facilitators and barriers to the implementation of an ICBT programme in a physical medicine rehabilitation setting from the perspective of clinicians, administrators, caregivers, and those with lived experience.

### Facilitators and barriers

Several factors were found to be facilitators for implementation of the ICBT programme in a physical medicine setting. Stakeholders with previous knowledge of the programme reported it to have *relative advantage*, *strong evidence and quality*, and *design quality*. The programme was also reported to have a *relative priority* as stakeholders perceived the ICBT programme to be central to meeting the mental health needs of patients. The effectiveness of the intervention and the accessibility of the online format has previously been reported to be important factors for its successful implementation (21, 35–37). For those with physical disabilities in particular, accessibility may be an important contributing factor for its implementation and adoption. Accessibility of the online model was reported by participants as a relative advantage as it may help improve the reach of mental health programmes across diverse groups of individuals with physical disabilities, as they may experience barriers such as geographical difficulties, lack of specialized services in their area, and lower socioeconomic status (21). Additionally, as those with physical disabilities often report comorbid complications such as cognitive impairments, fatigue, and/or physical impairments, implementing tailored ICBT programmes that meet the needs of this population is desirable. Previous studies have demonstrated that tailored ICBT programmes for those with disabilities can help to facilitate engagement and retention through the ability to (*i*) present materials in interactive and diverse formats (e.g., written, audio, video); (*ii*) visually adapt materials to aid with readability and comprehension; (*iii*) incorporate memory and planning aids, and (*iv*) remove the challenges associated with physically travelling to appointments (20, 21).

Several perceived barriers to implementation were found in the *inner setting*. These included *leadership engagement*, *culture*, and *available resources*. Leadership engagement has been identified as an important factor for successful implementation among several studies (38, 39). Previous studies have reported that establishing an organizational climate that emphasizes leadership engagement and promotes collaboration and flexibility can improve programme implementation (40, 41), as can ensuring that clinicians and administrators are engaged throughout the implementation process (42).

*Available resources* was the most commonly cited construct identified and related to lack of funding available for mental health services in a physical medicine setting. Though the participants stated that due to the nature of delivery, the programme could be seen as cost effective, i.e. requiring less therapist time and potentially resulting in cost savings per client, there was no funding available to integrate the services. Publicly funded allied healthcare services (such as physiotherapy and occupational therapy) may be available at the acute and subacute stage, but mental health services are often limited across the rehabilitation continuum from acute to community care (43). Strategies for seeking alternative forms of funding, such as mini-grants and donations or examining insurance models to subsidize programme delivery, have previously been suggested to promote sustainable patient access to evidence-based virtual programmes (44). Collaborations with community and not-for-profit organizations may also serve as alternative forms for programme delivery.

The barrier related to *adaptability* of the intervention was raised as a concern by clinicians in terms of potential lack of strategies for mitigating crisis interventions. These findings are consistent with previous research on implementing digital behavioural health programmes (21, 45–47). Among those with chronic health conditions, managing crises through a virtual care model may be a larger concern as these individuals are more likely to experience a higher rate of suicide than those in the general population. In order to better adapt and tailor interventions to meet the mental health needs of those with chronic health conditions undergoing physical rehabilitation, developing protocols for responding to crises after hours is important when planning to implement a virtual care model (16, 36, 48, 49).

Provider and user attitudes can influence implementation of an ICBT programme. These attitudes can include concerns from service providers around the creation and maintenance of a therapeutic alliance in a virtual environment affecting clinician buy-in. This could, in turn, affect the likelihood of clinicians referring clients to an online service. Differences in user comfort with digital technology in terms of literacy and *self-efficacy* in engagement in online programmes were also found to be potential barriers to implementation. Addressing concerns related to technology use and its effect on the therapeutic process is also recommended.^50^ Thus, sharing information with both providers and end-users around how easy the programme actually is to use may help persuade those who are hesitant to access the programme. Finally, developing more intuitive user interface designs for the web platforms used to deliver the programme, as well as simple step-by-step training videos, would allow users who are less familiar with technology to comfortably access the programme.

### Informing implementation and future directions

The current study has important implications for planning and developing implementation plans for ICBT in a physical medicine setting. A key component identified for implementation may be for leadership to foster an environment that involves collaboration and openness to change. Highlighting facilitators such as evidence-based practice, relative advantage, and relative priority of the ICBT programme may help facilitate buy-in from hesitant stakeholders. Developing ongoing workshops that provide information on the processes involved in safe and personalized delivery of the ICBT programme to those with greater needs may help to promote engagement among service providers. Involving those with lived experience and providers in the technology development process may help ease any concerns related to adaptability and usability of the intervention. Several studies have previously demonstrated the importance of end-user engagement in acceptability and adoptability of online technologies (50–54).

### Limitations and future directions

The current study is not without limitations. The current study utilized CFIR based on its previously discussed advantages. However, attempting to fit the provided feedback from participants into a pre-existing codebook may have led to additional themes not being explored in this study because they were not included in the CFIR codebook. Due to the qualitative nature of the study, it has the potential to be influenced by coder bias. The use of 2 independent reviewers, with a third coder being consulted when there were discrepancies, allowed us to minimize this bias. Several constructs of CFIR were not identified through the interviews in the current study. Previous studies have similarly found variations in the number of CFIR domains identified, with the number of domains found varying from 4 to 25 (21, 55), indicating that not all CIFR constructs may pertain to every implementation process. Relatedly, overlaps between applicable CFIR constructs created occasional challenges in reaching consensus in terms of response coding. Another limitation of the current study is that the CFIR model examines implementation from a unidirectional process, using a provider and administrator focus. Examination of the implementation process using a model that allows for a more multidimensional perspective, one that includes end-user engagement, might have been a better choice (50). However, examining end-user perspectives on ICBT service delivery and implementation in a physical medicine setting is lacking. The current study attempted to overcome this limitation through involvement of persons with lived experience and care partners. However, some constructs (e.g., inner setting) could not be examined through the end-user lens. Thus, expansion of implementation frameworks to evaluate more end-user perspectives is needed. The exploratory nature of the study, the convenience sampling, and the small sample size did not allow for examination of differences in perceptions among the various stakeholders. Examination of the perspectives of a wide range of persons with lived experience, including those with complex and varying degrees of impairments, is warranted. Lastly, the results may be impacted by previous experiences or lack thereof with ICBT. Thus, examining differences in perspectives of those with previous experience compared with those without may be important to develop strategies for optimal implementation planning.

Future research should aim to extend the current work in several respects. The results of this study can be used to develop an actionable implementation plan using Expert Recommendations for Implementing Change (ERIC) (56) in a physical medicine setting. Additionally, how ICBT services can be made scalable and better integrated with primary care is warranted. Examining various models for integration into the healthcare system as well as payment models is needed. Finally, ensuring legislative alignment with privacy and security of patient-level data when offering programmes across different regions is integral to ensuring adoptability.

### Conclusion

The current study provides insights on the potential facilitators and barriers to implementation of an ICBT programme in a physical medicine rehabilitation setting. Implementation is an active process that involves several factors for success. Provider and user beliefs concerning the effectiveness of the ICBT intervention and relative priority serve as facilitators. Barriers such as lack of leadership engagement, culture of inner setting, and available resources may serve as barriers. Leaders, service providers, and those with lived experience play an important role in facilitating the process (23,38). Thus, identifying ways for engaging them in the pre-implementation planning phase can be helpful to develop sustainable and integrated delivery of the ICBT programme. Although the CFIR framework was helpful in guiding the study, it was not without its limitations. Examining the implementation process via a more end-user framework may be warranted in the future.
